# Self-reported headache among the employees of a Swiss university hospital: prevalence, disability, current treatment, and economic impact

**DOI:** 10.1186/1129-2377-14-29

**Published:** 2013-03-26

**Authors:** Emina Sokolovic, Franz Riederer, Thomas Szucs, Reto Agosti, Peter Stefan Sándor

**Affiliations:** 1 Gilead Sciences Switzerland Sarl, Turmstrasse 28, Zug, CH-6300, Switzerland; 2 Department of Neurology, University Hospital Zurich, Zurich, CH-8091, Switzerland; 3 Baden Daettwil and RehaClinic (Zurzach, Baden, Braunwald, Glarus), Kantonsspital Baden, Baden, CH-5405, Switzerland; 4 Institute of Social and Preventive Medicine, University Zurich, Gloriastrasse 18a, Zürich, CH-8091, Switzerland; 5 Kopfwehzentrum Hirslanden Zürich, Forchstrasse 424, Zollikon, CH-8702, Switzerland

## Abstract

**Background:**

The objectives of this cross-sectional, observational study were to determine the prevalence of self-reported headache among the employees of the large Swiss university hospital, to measure the impact of headache using the MIDAS questionnaire, to assess current treatment and to estimate economic burden of headache considering indirect costs.

**Methods:**

A self-administered questionnaire was distributed internally to 2000 randomly selected employees of the University Hospital Zurich.

**Results:**

1210 employees (60.5%) responded. Of the 1192 (98.5%) employees who provided sufficiently complete information, 723 (61%) reported at least one headache type in the last three months. The prevalence of migraine, and tension-type headache was 20% and 50%, respectively. Regarding the occupational groups, there was a trend that healthcare staff, administration employees, and medical technicians suffered more from headaches than physicians, correcting for age and sex. The economic consequences of lost productivity were calculated to amount to approximately 14 million Swiss Francs (9.5 million EUR), representing 3.2% of the overall annual expenditure of the hospital for personnel.

**Conclusion:**

Headache is highly prevalent among university hospital employees, with significant economic impact.

## Background

Migraine is a common, chronic, potentially incapacitating neurovascular headache disorder, characterized by attacks of severe headache and autonomic nervous system dysfunction, with a substantial effect on economic productivity [1]. Physicians and patients often do not realize the migraine-associated disability, which may contribute to suboptimal management.

Primary headaches have been recognised by the World Health Organisation as major public health problem as they are globally prevalent in a considerable proportion of the population, affect all ages, and have significant impact on individuals and their society [2,3]. In the World Health Report 2001 [4] migraine figured among the top twenty causes of disability in the world, together with the neuropsychiatric disorders unipolar depressive disorders, alcohol use disorders, schizophrenia, bipolar affective disorder, Alzheimer’s and other dementias. Tension-type headache is usually less disabling than migraine, and typically lacking accompanying symptoms and has not yet been considered in WHO reports. The impact of this prevalent headache type on certain sufferers may be comparable to that of migraine, especially in those suffering from the chronic form. Importantly, from both migraine and tension headache, medication overuse headache (MOH) can arise, if acute headache medication is taken too often. MOH can phenotypically resemble tension-type headache, often with some migrainous features [5].

The socioeconomic significance of headache disorders is probably underestimated, in spite of some studies reporting their considerable impact on social activities and work [6]. The reduction in quality of life and work productivity due to migraine can be profound, with the intensity of pain being the most important of several factors. A Canadian study demonstrated that 77% of migraineurs have limitations of activity, 50% interrupt their activities and 30% must lie down during the attack [7,8].

Based on estimations of the global prevalence of active headache disorders [3] the number of tension-type headache and migraine sufferers in Switzerland should be around 3.2 million and 850 000, respectively [3]. In the Canadian population costs associated with migraine were estimated at 2229 Canadian dollars, whereby the major part of these expenses (1949 Canadian dollars) is attributable to indirect costs by absence from work and reduced productivity at work [9]. Applying these date to the Swiss population this would mean a total cost of 1.9 billion Canadian dollars (1.18 billion EUR, 1 EUR=1.6 Canadian Dollars in 2003) for migraine per year.

Despite the prevalence, severity, and burden of migraine, recent surveys suggest that fewer than half of current migraine sufferers have ever received a medical diagnosis [10]. Only one-third of migraine sufferers currently receive treatment with prescription drugs. The low rates of diagnosis and treatment have several causes, including low rates of medical consultation specifically for headache.

The main objectives of this cross-sectional observational study were to estimate (i) the prevalence of headache among employees of a large university hospital, (ii) the burden of headache -related disability using MIDAS (iii) economic implications by calculating the estimated indirect costs of headache.

## Methods

This is a cross-sectional survey of a sample of university hospital employees using a self-administered questionnaire. After approval of the Hospital Board and Ethics Committee, a questionnaire was sent to randomly chosen 2000 of 5525 employees of the university hospital from December 2002 to March 2003. This sample size was chosen prospectively assuming a response rate of 70% to allow confidence intervals of less than 5% either side of estimated headache prevalence. The hospital’s internal mail system was used for sending questionnaires and return envelopes. The study was announced on the university hospital intranet, a platform accessible to all employees, and a reminder to send back missing questionnaires was placed one month after the study start. Additionally, the announcement was placed in the show-case at the hospital entrance.

### Outcome measures

Participants were asked in a self-administered questionnaire if they had headache and / or migraine in the last 3 months. Those participants who responded with “yes” were asked to continue the 2 page questionnaire to provide information on:

• Diagnosis of migraine and tension-type headache, based on the criteria of the International Headache Society (IHS) [11]. Items related to headache diagnosis are presented in Table 1.

• headache days per month in the last 6 months

• family history of headache

• headache duration in years

• socio-demographic status (age, gender, occupation),

• consultation of headache specialists

• “double burden” of professional and family commitments,

• working hours per week

• impact on productivity at work, home and social activity as quantified with the migraine disability score (MIDAS) excluding the additional question B:

• On a scale of 0 – 10 on average how painful were these headaches [12,13].

**Table 1:** Items from headache questionnaires related to headache diagnosis

		
	**Type 1:**	**Type 2:**
a) Headache is	□ one-sided □ bilateral	□ one-sided □ bilateral
b) Headache is	□ pulsating/throbbing	□ pulsating/throbbing
	□ dull/pressing	□ dull/pressing
c) Daily activities are *impaired* (can still be performed) but not *inhibited* (cannot be performed anymore)	□ yes □ no	□ yes □ no
d) Headache worsened by physical activity	□ yes □ no	□ yes □ no
e) Nausea	□ yes □ no	□ yes □ no
f) Vomitting	□ yes □ no	□ yes □ no
g) Sensitivity to light	□ yes □ no	□ yes □ no
h) Sensitivity to noise	□ yes □ no	□ yes □ no
i) One or more completely reversible neurologic deficiencies (impaired vision or speech disorder)	□ yes □ no	□ yes □ no

### Assessment of the burden of headache with respect to disability and economic costs

Main outcome measures for headache related disability were workdays lost and reduced effectiveness at work and home due to headaches. The MIDAS score is the sum of missed days due to headache in the last three months from paid work, household work, non-work activities, plus days at paid work and in household work where productivity was reduced by at least half. The 4-point grading system for MIDAS Questionnaire is as follows: grade 1 (scores ranging from 0 to 5) = little or no disability; grade 2 (scores ranging from 6 to 10) = mild disability; grade 3 (scores ranging from 11 to 20) = moderate disability; grade 4 (score ≥ 21) = severe disability [14].

The average wages were calculated on the basis of the annual reports (average of 2000–2002 data) of the University Hospital according to the occupation, as stated in the questionnaire. The daily wages were calculated by dividing the annual income by 220 working days. The human capital approach was used for estimation of indirect costs [15], based on information from the MIDAS questionnaire (which covers the last 3 months).

### Statistical analysis

The questionnaire allowed respondents to be dichotomised as to whether they had or had not suffered from headache in the preceding 3 months. Up to two types of headaches could be reported and the number of patients with “at least” one type of headache was calculated, irrespective of the type. Headache prevalence was estimated in the sample (3 month prevalence). SPSS for Windows (version 10.0) was used to perform all the computations.

Continuous variables were expressed in descriptive terms, stating medians, means, interquartile ranges (IQR) and 95% confidence intervals. The assessment of univariate associations between candidate predictors and outcomes of interest was conducted as follows: (i) chi-square when exposures and outcomes were categorical, (ii) t-tests, Mann–Whitney-tests and analyses of variance resp. Kruskal-Wallis tests when one or more variables were continuous (depending on normality of data), (iii) correlation coefficients when both variables were continuous. Logistic regression was used to assess the association between outcome and several predictors, indicating odds ratios with 95% confidence intervals (CI). Two tailed p<0.05 was used as the level of statistical significance.

## Results

### Sample representativeness

**Figure 1:**
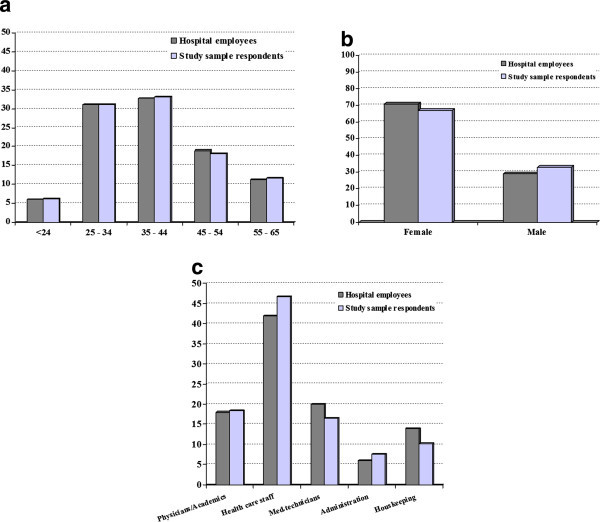
**Comparison of age (a), gender (b) and occupation (c) among the 1192 respondents and all university hospital employees (in %).** (**b**) Comparison of gender distribution among the 1192 respondents with those of University hospital employees (in %). (**c**) Comparison of distribution of occupation in the sample of 1192 respondents with the distribution of occupation of the university hospital employees in total (in %).

Of the 2000 randomly addressed employees who received the questionnaire, 1210 (60.5%) responded, but only 397 men and 795 women provided information on age and gender. The data from these 1192 respondents were analysed, though there were still omissions of information in some categories. As shown in Figures 1a - c., the distribution of age, gender and occupation amongst respondents to the questionnaire is comparable to the distribution amongst employees as a whole. Therefore, the study cohort can be viewed as representative for the population of the university hospital employees.

### Headache prevalence and socio-demographic variation

**Figure 2:**
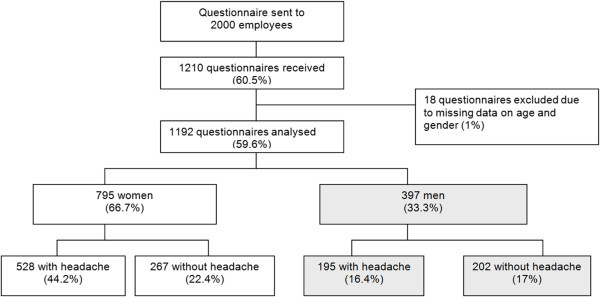
Flow chart of the study: Participation and sample characteristics.

Figure 2 shows a flow chart of the study with participation and sample characteristics. Of the 1192 employees who provided sufficiently complete information, 723 (61%) reported at least one headache type in the three months before completing the questionnaire. 292 (24%) of respondents reported more than one type of headache in the previous three months (227 females and 71 males). The 3 month prevalence was 20% (women 24%, men 13%) for migraine and 50% (women 55%, men 40%) for tension-type headache, considering both types of headache. Fourteen% of responders suffered from migraine and tension-type headache.

Median age of female and male respondents suffering from migraine or tension-type headache was not significantly different (Table 2). Participants who did not report having headache were somewhat older (median 40 years, IQR 34–48), though not significantly. The majority of the female headache sufferers were in the youngest age-group 18–35 (45%), followed by 39% aged between 36 and 50 years, whereas only 16% of female headache sufferers were 51 and older. Half of the male headache sufferers were in the age group between 36 and 50 years (50%), followed by 36% in the youngest group and 14% male headache sufferers were 51 and older.

**Table 2:** Median age among different types of headache and gender (n=1192)

**Main headache type median (IQR)**			
**Gender**	**Migraine**	**Tension**	**Other**
Female	38 (31–46)	37 (29–44)	35 (30–42)
Male	38 (32–50)	38 (34–45)	37 (29–43)

The inter quartile ranges (IQR) are given in brackets.

**Figure 3:**
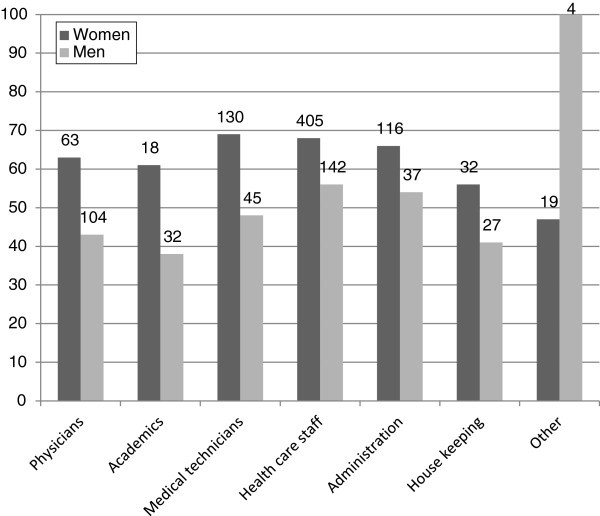
**Percentage of employees with headache distributed according to occupational groups.** After correction for age and sex there was at trend that healthcare staff, administration, and medical technicians suffered more from headaches than physicians. See text. The absolute numbers of respondents are given on the respective columns.

In absolute numbers, the health care-staff suffered more from headaches than any other occupational group (Figure 3). Logistic regression revealed a strong association with age (p<0.001) and sex (p<0.001). There was a trend that healthcare staff, (OR 1.51, 95% CI 1.05-2.2, p=0.028), administration employees (OR 1.61, 95% CI 1.00-2.60, p=0.048) and medical technicians (OR 1.50 95% CI 0.95-2.63, p=0.080) suffered more from headaches than physicians; however these trends did not survive correction for multiple comparisons.

Median monthly frequency of type 1 headache in the preceding 6 months was 3 days (IQR 2–6). There was a trend towards higher frequencies in the older age group, especially in individuals with other types of headache. However, this was not statistically significant (Table 3).

**Table 3:** Monthly frequency of main headache type among age categories and gender in the the preceding six months (n=1192)

		**Main headache type median (IQR)**		
		**Migraine**	**Tension**	**Other**
Age category	18 – 35	3 (2–5)	3 (2–6)	3 (1–6)
	36 – 50	4 (2–6)	3 (2–5)	2 (2–5)
	51 – 65	5 (2–8)	3 (2–5)	8 (3–10)
Gender	Female	4 (2–6)	3 (2–5)	5 (2–7)
	Male	4 (2–7)	3 (2–5)	2 (1–5)

A family history of headache was reported by 428 (59%) of all headache sufferers, mainly females (76%).

The median time since onset of headache was 10 years (IQR 5–20), with only little gender differences. A total of 295 headache sufferers (42%) reported a double burden of having family as well as professional commitments with no significant differences between women (224/513; 44%) and men (71/191; 37%; p=0.123).

### Medical resources used and medication

Only 27% of the employees, who reported headache, had ever consulted a physician, mainly a general practitioner. Only 4% of women and 2% of men had ever consulted a neurologist. (Table 4). As expected, the individuals with higher headache frequency (p=0.009), and higher disability scores (p=0.001), showed higher tendency to consult the physicians.

**Table 4:** Physician consultations in headache sufferers

**Physician consulted**	**Women**	**Men**	**Total**
	**N (%)**	**N (%)**	**N (%)**
General practitioner	98 (9)	33 (17)	131 (18)
Internist	16 (3)	7 (4)	23 (3)
Neurologist	21 (4)	3 (2)	24 (3)
Other	15 (2)	5 (3)	20 (3)
None	375 (71)	147 (75)	522 (73)

About one third of headache sufferers used no medication. Over the counter drugs were used most frequently, specific migraine medications such as triptans or prophylaxic medication only by a small minority (Table 5).

**Table 5:** Medication use in headache sufferers

**Main headache type N (%)**				
**Medication**	**Migraine**	**Tension**	**Other**	**Headache**
Women				
No medication	40 (23)	121 (40)	9 (25)	170 (34)
OTC*	72 (42)	120 (40)	23 (63)	215 (42)
Prescription	49 (28)	55 (19)	3 (8)	107 (21)
Triptans	8 (5)	2 (1)	1 (3)	11 (2)
Prophylaxis	3 (2)	0	0	3 (0)
Men				
No medication	7 (16)	32 (29)	12 (40)	51 (28)
OTC*	22 (49)	56 (51)	12 (40)	90 (49)
Prescription	10 (22)	21 (19)	5 (17)	36 (20)
Triptans	4 (9)	0	0 (0)	4 (2)
Prophylaxis	2 (4)	0	1 (3)	3 (2)

* Over the counter.

### Disability due to headache and economic impact

**Figure 4:**
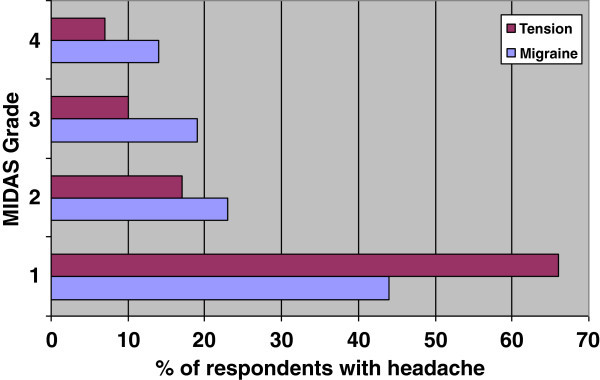
**MIDAS grades in respondents with migraine and tension-type headache employed in a Swiss university hospital.** Grade 1 (scores 0–5) = little or no disability; grade 2 (scores 6–10) = mild disability; grade 3 (scores 11–20) = moderate disability; grade 4 (score ≥ 21) = severe disability [14]. There was significant association between headache diagnosis and MIDAS grade (p<0.001).

Median MIDAS score in headache sufferers was 4 (IQR 1–10). The MIDAS grades in respondents with migraine and tension-type headache are shown in Figure 4. There was significant association between headache diagnosis and MIDAS grade (p<0.001).

In regression analyses, association of each variable with MIDAS score was examined. Significant independent associations with the MIDAS score were observed for headache frequency, family history, double burden, medication- use, number of working hours per month and physician consultation. These variables explained 16% of the variation of the MIDAS score (p=0.001; r [2]=0.16). None of the other variables explained variability in MIDAS score. This was unexpected for demographic characteristics.

Over 60% of all headache sufferers reported to be full-time employed, the rest part-time. Mean number of workdays lost due to headache in the previous 3 months was 0.65 (95% CI: 0.57-0.73) in headache sufferers. The reported mean reduction of productivity by 50% in the preceding three months because of headaches was 2.2 days (95% CI: 1.97; 2.49).

The individuals with migraine/headache reported, that the mean number of days, on which they were not able to do housework, was 1.60 in the previous three months (95% CI: 1.39; 1.89). Household work was reduced by 50% on 2.12 days (95% CI: 1.86; 2.38), and social/leisure activities were missed on 2.37 days (95% CI: 2.07; 2.67) on average in the preceding three months.

Extrapolation of patient self-reported work and productivity loss for the last three months to an annual basis suggested that individuals with headaches lose 10.2 workday equivalents per year (2.4 days from absenteeism, 7.8 days with ≥ 50% loss in productivity while at work, counted as full days lost, ignoring days with < 50% productivity loss [16]). Taking the average wages of the headache sufferers (CHF 408.4/person/day) into account, the annual costs of lost labour days was estimated at CHF 980.18 (662.28 EUR; 1 EUR=1.48 CHF in 2003) whereas the annual loss of productivity accounts for another CHF 3^′^185.60 (2^′^152.43 EUR). On the basis of the results obtained, and assuming that the study sample is representative of the employees as a whole (N=5525), it is possible to estimate the overall economic impact on the university hospital based on the average days off work in employees with headache and the prevalence of headache (61%). The economic consequences of this productivity loss may thus equal approximately 14 million Swiss Francs (9.5 million EUR), representing 3.2% of the overall annual personnel expenditure of the hospital.

## Discussion

The three month prevalence of self-reported headache was 61% in this hospital-based sample. The high prevalence of migraine in our sample (20%) can be explained at least in part, by the fact that about 70% of employees were female (Figure 1b). Furthermore, age distribution in the workplace setting of our study (Figure 1b) is probably shifted towards younger ages, where migraine is more prevalent compared to the general population. Finally, the high prevalence of migraine the hospital setting gives rise to speculations that this is a particularly stressful environment since stress is a frequent trigger factor for migraine attacks [17]. Taking into account the influence of sex and age, there was a trend towards higher headache prevalence among the health care staff, administration employees, and medical technicians, indicating that these work fields might be particularly demanding.

In accordance with previous studies, we found a female preponderance for migraine and tension-type headache and lower prevalence in older age groups [18,19].

Less than one third of headache sufferers in our sample had consulted physicians for their headaches. We found that even in the hospital environment that offers plenty of medical facilities, headache specialists, such as neurologists, are consulted only rarely. A significant proportion of headache and migraine sufferers had only consulted primary care physicians. This is in accordance with previous studies [20-22]. Also in accordance with previous evidence [23-25] only a minority of migraine sufferers used migraine-specific agents (triptans) or prophylactic medication in our sample.

The economic impact of migraine and other headache-associated disorders can be considered in terms of the direct and indirect costs. The *direct* costs are those related to the costs of the medications and the time of any healthcare personnel involved in treatments (most of which are estimated arbitrarily in the form of fixed reimbursement fees); because such a large proportion of migraine sufferers do not even register in any account of healthcare activity, the overall burden of direct costs is almost impossible to estimate [26]. The *indirect* costs are those associated with absence from work and reduced effectiveness while at work. Because prevalence of migraine peaks during the most productive years of life, between ages of 25 and 55, it is an important cause of lost work time. Indirect costs account for more than 80% of economic burden [27,28]. We estimated the economic consequences of productivity loss (*indirect* costs) related to headache in the university hospital to 14 million CHF (9.5 million EUR), representing 3.2% of the overall annual personnel expenditure. Annual indirect costs per headache sufferer (CHF 4165.68 = EUR 2814.72) were higher than the costs of migraine in Europe, which were estimated at EUR 1222 (93% of this amount are indirect costs) in a recent study [29]. The discrepancy may be explained by the populations studied (work place setting versus general population) and differences in wage levels.

Distribution of MIDAS grades was different between migraine and tension-type headache: More tension-type headache sufferers had MIDAS grade 1, while more migraine sufferers had MIDAS grades 2–4. We found a positive correlation of missed days of work and days with decreased productivity with the increased grade of MIDAS score. MIDAS, a measure of headache related disability, was associated with double burden (professional and family commitments) and number of working hours per month in addition to indicators of migraine severity such as headache frequency, medication use or physician consultation.

Several shortcomings of our study merit discussion. First, the 60.5% responder rate may address the question of participation bias. Although, representativeness of the sample was shown, as the responders and non-responders distribution with respect to age and gender were comparable, participation bias cannot be fully excluded. As the focus of the study was headache related disability and cost in general, we only considered headache as such for economic estimations, irrespective of type. Therefore possible diagnostic problems of self-administered questionnaires can be considered negligible in this context. Conclusions from the present study conducted in the environment of a large university hospital may not be comparable to other workplace settings with different organisation of work and wage levels.

Finally, a limitation of our study is the use of the human capital method, according to which production losses are valued using average earnings, whereas actual loss to society may be much smaller. Migraine and tension type headache led to short-time absences that may not result in production loss: the work may be covered by others or made up by the headache sufferer on his return to work.

The strengths of the study include the large sample size.

Population-based studies reveal that migraine is currently under-diagnosed and under-treated as a whole, but estimation of the distribution and magnitude of the impact of headache in the workplace is necessary before public health interventions can be developed.

## Conclusion

In conclusion, headache is a highly prevalent condition among university hospital employees, with significant impact on both, professional and social functioning. There is a substantial economic burden of headache from an employer perspective. Our findings suggest that effective diagnosis and treatment of migraine could contribute substantially to reduce the indirect cost associated with this disabling disorder.

## Competing interests

The authors declare that they have no competing interests.

## Authors’ contributions

PS and RA designed the study and corrected the manuscript. ES collected and analysed the data and wrote a draft of the manuscript. FR analysed the data, corrected the manuscript and wrote parts of it. TS designed the study. All authors read and approved the final manuscript.
